# *PE* (*Prickly Eggplant*) encoding a cytokinin-activating enzyme responsible for the formation of prickles in eggplant

**DOI:** 10.1093/hr/uhae134

**Published:** 2024-05-10

**Authors:** Lei Zhang, Runzhi Zhang, Ping Yan, Liqian Zeng, Weiwei Zhao, Huiqian Feng, Ruyu Mu, Wenqian Hou

**Affiliations:** Jiangsu Key Laboratory of Phylogenomics and Comparative Genomics, Jiangsu International Joint Center of Genomics, School of Life Sciences, Jiangsu Normal University, Xuzhou 221100, Jiangsu Province, China; Jiangsu Key Laboratory of Phylogenomics and Comparative Genomics, Jiangsu International Joint Center of Genomics, School of Life Sciences, Jiangsu Normal University, Xuzhou 221100, Jiangsu Province, China; Jiangsu Key Laboratory of Phylogenomics and Comparative Genomics, Jiangsu International Joint Center of Genomics, School of Life Sciences, Jiangsu Normal University, Xuzhou 221100, Jiangsu Province, China; Jiangsu Key Laboratory of Phylogenomics and Comparative Genomics, Jiangsu International Joint Center of Genomics, School of Life Sciences, Jiangsu Normal University, Xuzhou 221100, Jiangsu Province, China; Jiangsu Key Laboratory of Phylogenomics and Comparative Genomics, Jiangsu International Joint Center of Genomics, School of Life Sciences, Jiangsu Normal University, Xuzhou 221100, Jiangsu Province, China; Jiangsu Key Laboratory of Phylogenomics and Comparative Genomics, Jiangsu International Joint Center of Genomics, School of Life Sciences, Jiangsu Normal University, Xuzhou 221100, Jiangsu Province, China; Institute of Vegetables and Flowers, Chinese Academy of Agricultural Sciences, Beijing 100081, China; Jiangsu Key Laboratory of Phylogenomics and Comparative Genomics, Jiangsu International Joint Center of Genomics, School of Life Sciences, Jiangsu Normal University, Xuzhou 221100, Jiangsu Province, China

## Abstract

Eggplant is one of the most important vegetables worldwide, with some varieties displaying prickles. These prickles, present on the leaves, stems, and fruit calyxes, posing challenges during cultivation, harvesting, and transportation, making them an undesirable agronomic trait. However, the genetic mechanisms underlying prickle morphogenesis in eggplant remain poorly understood, impeding genetic improvements. In this study, genetic analyses revealed that prickle morphogenesis is governed by a single dominant nuclear gene, termed *PE* (*Prickly Eggplant*). Subsequent bulk segregant RNA-sequencing (BSR-seq) and linkage analysis preliminarily mapped *PE* to chromosome 6. This locus was then fine mapped to a 9233 bp interval in a segregating population of 1109 plants, harboring only one candidate gene, *SmLOG1*, which encodes a LONELY GUY (LOG)-family cytokinin biosynthetic enzyme. Expression analyses via transcriptome and qRT-PCR demonstrate that *SmLOG1* is predominantly expressed in immature prickles. CRISPR-Cas9 knockout experiments targeting *SmLOG1* in prickly parental line ‘PI 381159’ abolished prickles across all tissues, confirming its critical role in prickle morphogenesis. Sequence analysis of *SmLOG1* pinpointed variations solely within the non-coding region. We developed a cleaved amplified polymorphic sequences (CAPS) marker from a distinct SNP located at −735-bp within the *SmLOG1* promoter, finding significant association with prickle variation in 190 eggplant germplasms. These findings enhance our understanding of the molecular mechanisms governing prickle development in eggplant and facilitate the use of marker-assisted selection (MAS) for breeding prickleless cultivars.

## Introduction

Eggplant (*Solanum melongena* L.) ranks as the third most significant crop within the *Solanaceae* family, surpassed only by potatoes and tomatoes [1]. Unique among Solanaceous crops of the Old World, the eggplant has been subjected to an extensive domestication process [2] and is distinguished by its prickle phenotype [3]. Prickles emerge across different plant tissues, including leaves, stems, and calyxes, acting as a defense mechanism against herbivores and physical harm [4]. Nevertheless, the presence of prickle is generally deemed adverse, potentially complicating the agricultural practices, including cultivation, harvesting, and transportation [3]. Consequently, the cultivation of prickleless eggplant varieties has become a critical goal in breeding programs.

Prickles, commonly found in plant genera like *Caesalpinia*, *Rubus*, *Rosa*, and *Solanum*, are pointed, hardened structures that arise from the surface layers, such as the epidermis or the layers beneath it, and lack vascular tissues, making them easily detachable [5]. In contrast, thorns and spines, which are deeply embedded and vascularized modifications of plant stems or leaves, remain securely attached [5, 6]. Similar to prickles, trichomes also originate from the epidermal tissue of plants [7]. Intriguingly, research has revealed potential overlaps between the molecular mechanisms governing the development of trichomes and prickles in various plants, including grape [8], roses [9], and *Rubus* [10, 11]. Nevertheless, emerging studies propose that the formation of prickles might follow unique genetic pathways, distinct from those of trichomes [12–15]. For instance, in rose species, research indicates that prickles develop from multiple cells originating from the ground meristem below the protoderm [12, 16], which contrasts with the trichomes’ typical development from one or several cells of the protoderm (or epidermis) only [17]. Additionally, gene expression analyses have revealed that genes associated with trichome initiation do not show the same expression patterns during prickle development in roses [13]. Despite a growing interest, functional studies on prickle regulating genes are notably scarce. This lack of definitive research contributes to the ongoing ambiguity surrounding the genetic basis of prickle formation. Therefore, identifying and analysing the key genes responsible for prickle formation is essential for shedding light on the mechanisms underlying their morphogenesis.

To unravel the genetic mechanisms responsible for the emergence of prickly traits in crops like eggplant and rose, extensive genetic research has been conducted [13, 18]. Despite the progress, pinpointing the key genes responsible for prickle development remains a challenge. In eggplant, the prickle phenotype is a complex trait governed by multiple quantitative trait loci (QTL), as has been identified in diverse populations. These QTLs, associated with prickles on the leaf, stem, and calyx, have been mapped to all chromosomes except 10 and 11 [3, 19–25]. Notably, a major QTL related to calyx prickle development was located within a 7 kb interval on chromosome 12, identifying a gene encoding a WUSCHEL-related homeobox-like protein as the candidate [25]. Another QTL on chromosome 6 responsible for prickle presence or absence was fine-mapped to an interval of 133-kb, leading to the development of a PAV marker to assist in breeding programs [3]. Furthermore, this QTL was narrowed down to a 28.3 kb region, identifying *SmARF18*, an auxin response factor, as a potential candidate due to a non-synonymous SNP [18]. However, neither of these candidate genes has undergone functional validation to confirm their role in prickle formation. Additionally, no loss-of-function mutations were detected in the coding region of *SmARF18* in our studied populations. Thus, it suggests that prickle morphogenesis in eggplant may involve additional genes. Further exploration of other genetic elements is required to fully elucidate the complexity of prickle formation in eggplant and to identify potential targets for breeding prickleless varieties.

In this study, we dissected the genetic basis of prickle development through analysis of divergent populations derived from a prickleless inbred line ‘XQ23’ and a prickly line ‘PI 381159’. Our findings revealed that prickle formation is controlled by a single dominant nuclear gene, named *PE* (*Prickly Eggplant*). By combining BSR-seq and linkage analysis, the *PE* gene was localized to a 9233 bp interval on chromosome 6, identifying *SmLOG1* as the candidate gene. This gene encodes a *Lonely Guy* (*LOG*) homologous enzyme essential for the final step in cytokinin synthesis. The knockout of *SmLOG1* in the prickly parent via CRISPR-Cas9 resulted in a complete loss of prickles, validating its essential role in prickle development. Sequence analysis revealed that a specific SNP in its promoter region was significantly associated with prickle variance among 190 eggplant germplasms. This association enabled the development of a cleaved amplified polymorphic sequences (CAPS) marker to support marker-assisted breeding efforts. These results deepen our understanding of the molecular pathways that control prickle formation in eggplant and pave the way for employing marker-assisted selection (MAS) to develop prickleless eggplant varieties.

## Results

### Morphological characterization and inheritance of prickle in eggplant

**Figure 1:**
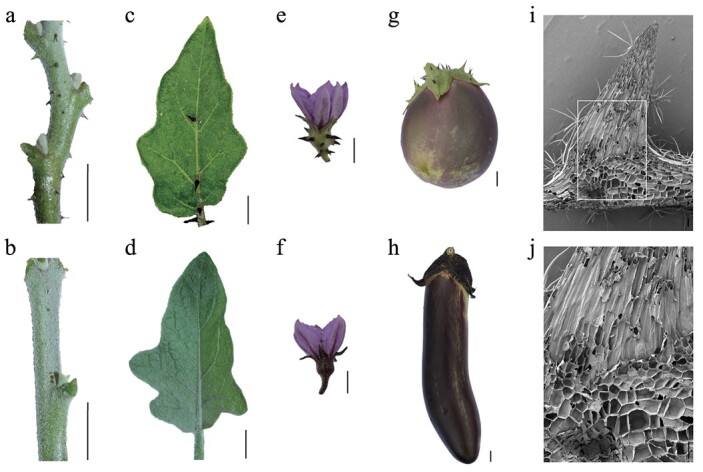
Morphological characterization of prickle in eggplant. **a**–**h** Phenotype comparison between two parental lines in different tissues: stems (**a**, **b**), leaves (**c**, **d**), flowers (**e**, **f**), and fruits (**g**, **h**) from the prickly eggplant ‘PI 381159’ and the prickleless eggplant ‘XQ23’, respectively. Black bars represent scales of 1 cm. **i** Scanning electron micrographs of longitudinally sliced prickles. **j** Magnified representation of the section highlighted by the white square in graph, illustrating the junction between the prickle and the stem epidermis. Black bars represent scales of 1 mm (**i**, **j**).

Prickle characteristics were visually compared between two parental lines. It was observed that prickles are present on the stems, leaf veins, flower calyces, and fruit calyces of the prickly parent ‘PI 381159’, whereas the prickleless eggplant parent ‘XQ23’ lacked prickles in these tissues (Fig. 1a–h). Further examination of the ‘PI 381159’ stem surface with a scanning electron microscope showed branched trichomes interspersed with prickles (Fig. 1i). At the intersection between the prickle and stem epidermis, the cells comprising the prickle resembled those of the stem’s epidermis in shape and size, suggesting that prickles are outgrowths of the epidermis. Additional analysis of the prickles showed that they are multicellular structures, lacking both vascular bundles and glandular structure (Fig. 1i and j).

Genetic analysis of the prickle phenotype was performed across three populations (F_2_, BC_1_P_1_, BC_1_P_2_), with the segregation ratios detailed in Table S1 (see online supplementary material). Analysis revealed that the allele for prickles is dominant over the allele for pricklelessness. The chi-square (χ2) test demonstrated that the segregation ratios in the F_2_ and BC_1_P_1_ populations adhere to 3:1 and 1:1, respectively. This suggests that a single dominant nuclear gene, designated as *PE* (*Prickly Eggplant*), governs the prickle phenotype in eggplant.

### BSR-seq analysis and preliminary mapping of the *PE* gene

**Figure 2:**
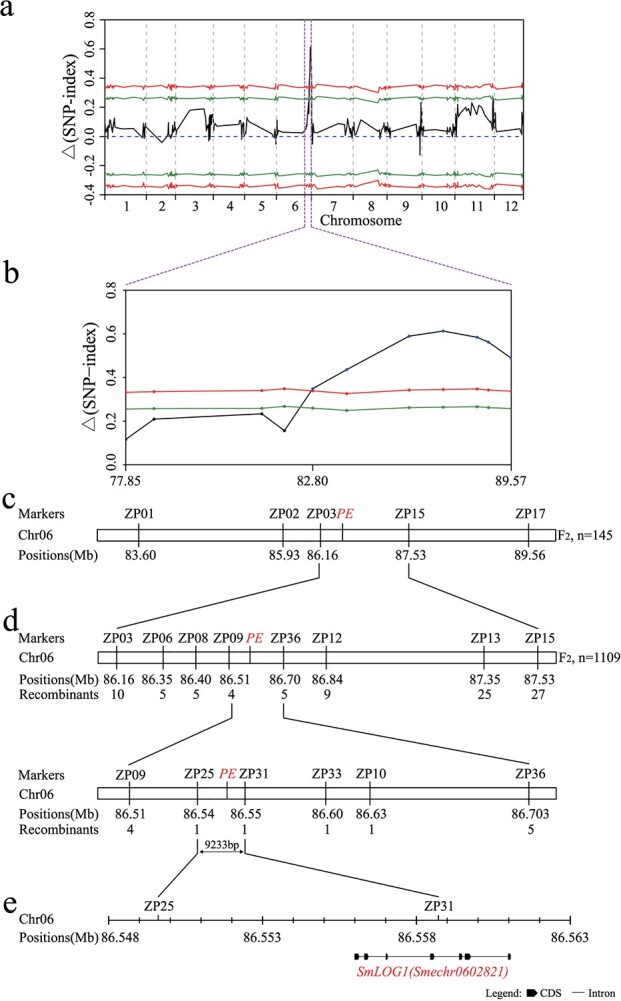
Map-based cloning of *PE*. **a**  *△*(SNP-Index) values plotted along the eggplant genome. The green and red curves represent the statistical confidence intervals, corresponding to the null hypothesis that there are no quantitative trait loci, at significance levels of *P* < 0.05 and *P* < 0.01, respectively. **b** The locus ranging from 82.80 to 89.57 Mb on chromosome 6, with a significance level of *P* < 0.01, has been identified to control eggplant prickle development. **c** Preliminary mapping. The *PE* was located between markers ZP03 and ZP15. **d** Fine mapping. The *PE* was mapped to a 9233 bp region between markers ZP25 and ZP31. **e** Detailed schematic representation illustrating the gene structure of the candidate gene, *SmLOG1*.

BSR-seq approach was utilized for the initial mapping of the *PE* gene. For the F_2_ segregating population, separate pools for prickly and prickleless phenotypes were established, and both were subjected to RNA-seq, yielding 6.69 Gb and 6.74 Gb of data, respectively (Table S2, see online supplementary material). The sequencing data were aligned with the eggplant reference genome HQ-1315, single nucleotide polymorphism (SNP) was identified and the *Δ*(SNP-Index) for each SNP locus, along with their 95% and 99% confidence intervals, were computed. These values were averaged in 400 kb windows with a step size of 200 kb. As shown in Fig. 2a, a significant locus was identified at the end of chromosome 6. Further analysis of this locus revealed significantly linked regions extending from 82.80 to 89.57 Mb at the 99% confidence interval (*P* < 0.01), indicating the probable location of the *PE* gene (Fig. 2b).

To confirm the *PE* locus identified via the BSR-seq approach, a small population consisting of 145 F_2_ individuals was employed for linkage analysis. Five CAPS markers designed within the candidate region (Table S3, see online supplementary material) were used to screen the segregating population. As a result, the gene was preliminarily mapped between markers ZP03 and ZP15, within an interval of 1.37 Mb between 86.16 to 87.53 Mb on chromosome 6 (Fig. 2c).

### Fine mapping of *PE* to a 9233 bp interval

To refine the candidate region further, an additional 1109 individuals from the F_2_ segregating population were utilized for the fine mapping of the *PE* gene. The markers ZP03 and ZP15 were employed to screen this population, which led to the identification of 37 recombinants. Subsequently, eight CAPS markers and two SNP-based sequencing markers were developed to genotype these recombinants (Table S3, see online supplementary material). The analysis delimited the candidate region to the interval between markers ZP25 and ZP31, each closely linked to the *PE* gene, with each marker exhibiting only one recombinant. Progeny testing was subsequently performed to validate the genotypes of these two recombinants based on the segregation observed in their offspring’s phenotypes. Consequently, the *PE* gene was pinpointed to the region between markers ZP25 and ZP31 (Fig. 2d). According to the eggplant genome sequence, the physical distance between these two markers is 9233 bp.

### Identification of *SmLOG1* as the candidate gene for *PE*

Based on the eggplant genome annotation, there is no fully annotated gene present within the 9233 bp interval. However, a partial region of a gene named *Smechr0602821* is included in this interval (Fig. 2e). The *Smechr0602821* gene is composed of seven exons and has a coding sequence (CDS) of 618 bp, which encodes 205 amino acids. BLAST alignment against the Arabidopsis genome revealed that Smechr0602821 exhibits the highest amino acid sequence identity (80%) with ATLOG1, a member of the *Lonely Guy* (*LOG*) gene family. Thus, we named *Smechr0602821* as *SmLOG1*. Previous studies have revealed that *LOG* genes encode cytokinin riboside 5′-monophosphate phosphoribohydrolases. These essential enzymes convert inactive cytokinin nucleotides directly into active cytokinin free bases, a key step in cytokinin synthesis. This function is pivotal for the maintenance of meristem regions, significantly impacting the plant’s overall development and response to environmental stimuli [26–28]. Consequently, we propose that *SmLOG1* might play a role in the regulation of prickle development.

**Figure 3:**
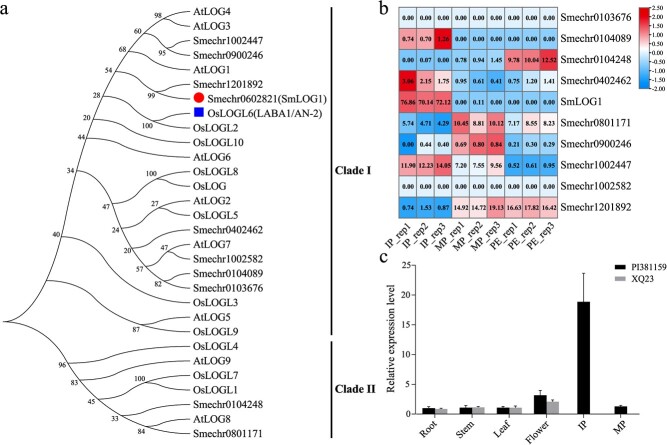
Phylogenetic and expression analysis of *SmLOG1*. **a** Phylogenetic analysis of LOG proteins from *Arabidopsis thaliana*, *Solanum melongena* (eggplant), and *Oryza sativa* (rice), denoted by the prefixes At, Os, and Sme, respectively. Bootstrap values are indicated at the branching points. **b** Heatmap depicting the expression patterns of eggplant *LOG* genes in immature prickles, mature prickles, and prickleless epidermis. The heatmap is based on normalized log_2_(FPKM+1) values. Each tissue type was represented by three biological replicates. The FPKM values are shown in each cell. **c** Expression profiles of *SmLOG1* across various tissues using qRT-PCR. In (**b**) and (**c**), IP, MP, and PE correspond to immature prickles, mature prickles, and prickleless epidermis, respectively.

To comprehensively characterize LOG proteins in eggplant, we utilized the amino acid sequences of SmLOG1 and its homologs in Arabidopsis and rice as queries to BLAST against the eggplant genome. This approach led to the identification of 10 LOG proteins in eggplant (Table S4, see online supplementary material). Subsequently, we constructed a phylogenetic tree for the LOG proteins from eggplant, Arabidopsis, and rice. The phylogenetic tree revealed that the LOG proteins could be categorized into two distinct clades: clade I and clade II (Fig. 3a). It was observed that eight SmLOG proteins, including SmLOG1, were classified under clade I. Within this clade, OsLOGL6 (LABA1/An-2) has been reported to regulate the formation of barbs and the elongation of awns [29, 30]. Barbs are small, hook-like structures found on the awns, which are single cells that originate from epidermal cells [29]. Thus, like prickles, barbs are also outgrowths of the epidermis. Based on the evidence provided, we propose *SmLOG1* as a candidate gene for *PE*.

It is noteworthy that SmLOG1 and Smechr1201892 cluster together in the same branch, underscoring their close genetic relationship. This clustering is supported by our synteny analysis, which revealed that the two genes are located within syntenic blocks that likely originated from a segmental duplication event (Fig. S1, see online supplementary material). The sequence alignment shows a high level of similarity between the two genes, with an amino acid sequence identity of 83.5%. Further sequence analysis revealed significant differences in their promoter and intron regions, implying that these genes may have experienced functional subdifferentiation following a duplication event (Fig. S2, see online supplementary material). It is reported that the spatially specific expression of *LOG* genes dictates cytokinin distribution, influencing the activity of meristematic tissues and consequently affecting the development of plant organs [31]. In plants like *Arabidopsis*, distinct members of the *LOG* gene family have been shown to control different aspects of organ development [27, 28]. We suggest that the observed variations in the promoter regions are likely contributing to the functional divergence between these genes.

### Predominant expression of *SmLOG1* and cytokinin signaling-related genes in immature prickles

Utilizing transcriptomic data from our previous studies, it was observed that the expression level of *SmLOG1* was significantly higher in immature prickles compared to mature prickles or prickleless epidermis (Fig. 3b; Table S5, see online supplementary material). We subsequently analysed the expression patterns of *SmLOG1* in various tissues, including stem, leaf, root, flower, immature prickle, and mature prickle, between the prickly parent ‘PI 381159’ and the prickleless parent ‘XQ23’ by employing qRT-PCR (Fig. 3c). The results indicated that *SmLOG1* displayed the highest expression level in the immature prickles of the prickly parent ‘PI 381159’. Conversely, the prickleless parent ‘XQ23’ showed low expression levels across all tissues. Combined with the transcriptome and qRT-PCR data, it is suggested that *SmLOG1* was predominantly expressed in the immature prickles, highlighting its significant role in the morphogenesis of prickles.

Given the specific expression of *SmLOG1* in immature prickles, we explored the potential involvement of the cytokinin signaling pathway in prickle development. This pathway comprises histidine kinase receptors (HKs), histidine phosphotransfer proteins (HPs), and response regulators (RRs) [32, 33]. Using Arabidopsis cytokinin signaling-related genes as references, we identified 54 homologous genes in the eggplant genome through homology searches (Table S6, see online supplementary material). Several key genes within this pathway, including *Smechr1102258* (*Histidine Kinase Receptor 4*, *HK4*), *Smechr0101117* (*Histidine Phosphotransfer Protein 1*, *HP1*), *Smechr0301654* (*Response Regulator 4*, *RR4*), *Smechr1002795* (*Response Regulator 9*, *RR9*), and *Smechr1002833* (*RR9*), exhibited significantly higher expression in immature prickles than in mature prickles or prickleless epidermis (Fig. S3, see online supplementary material). Their expression patterns are consistent with that of *SmLOG1*, suggesting that cytokinin signaling plays a critical role in prickle development.

### Knockout of *SmLOG1* abolished prickles

**Figure 4:**
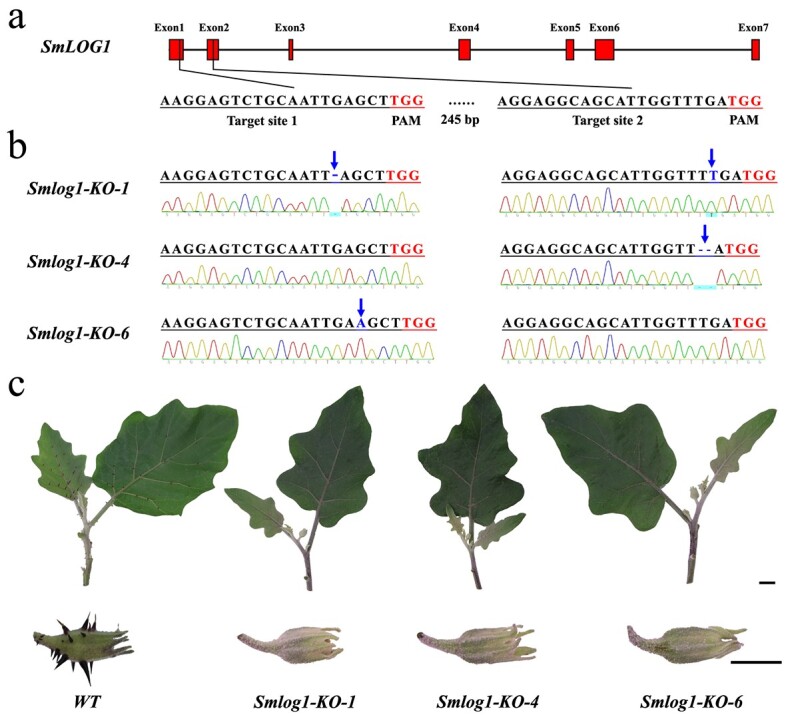
CRISPR-Cas9-induced mutations in eggplant. **a** Schematic diagram of the two guide sequences targeting the first and second exons of *SmLOG1*. The target sequences are shown in black, while the protospacer adjacent motif (PAM) are highlighted in red. **b** Direct sequencing of PCR product chromatograms from the target region of *SmLOG1* in three representative homozygous T_1_ plants. Blue arrows highlight mutation sites, with blue lines or nucleotides showcasing deletion or insertion mutations. **c** Phenotypes of three *SmLOG1* knockout mutants (*Smlog1-KO-1*, *Smlog1-KO-4*, and *Smlog1-KO-6*). Scale bar, 1 cm.

To confirm the role of *SmLOG1* in prickle development, we constructed a CRISPR-Cas9 vector containing two sgRNAs targeting the coding region (Fig. 4a). We then introduced this vector into the prickly parent line ‘PI 381159’ via Agrobacterium-mediated transformation and obtained 12 T_0_ transgenic plants. Following this, we amplified and sequenced the genomic regions containing the target sites in each T_0_ transgenic plant to evaluate the mutations introduced into *SmLOG1*, leading to the identification of InDel or SNP mutations in the gene. We then self-crossed the T_0_ transgenic plant to obatin three T_1_ homozygous knockout mutants. Specifically, the CRISPR line *Smlog1-KO-1* contained a 1 bp deletion in the first target site and 1 bp insertion in the second target site, *Smlog1-KO-4* contained only a 2 bp deletion in the second target site, and *Smlog1-KO-6* contained 1 bp insertion in the first target (Fig. 4b). Moreover, Sanger sequencing was performed to confirme that there were no mutations at the *Smechr1201892* locus, which shares high similarity with *SmLOG1*, in any of the above three T_1_ homozygous knockout mutants (Fig. S4, see online supplementary material). Notably, the T_1_ homozygous knockout mutants exhibited a complete absence of prickles across all tissues, confirming the pivotal role of *SmLOG1* in prickle morphogenesis. However, the formation of trichomes on these mutants was not affected (Fig. 4c; S5, see online supplementary material). These findings collectively establish *SmLOG1* as the correct candidate gene for the *PE*.

### A SNP within the *SmLOG1* promoter significantly associated with variations in prickle trait in eggplant germplasms

**Figure 5:**
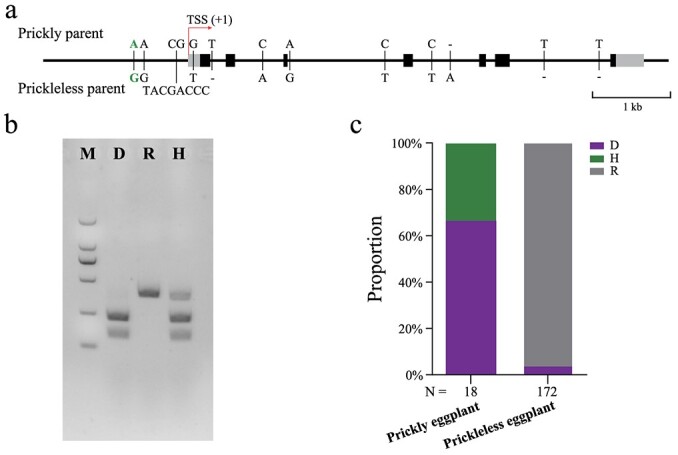
Analysis of sequence variations in *SmLOG1*. **a** Schematic representation of the structure and sequence variations of *SmLOG1*. The grey boxes represent UTRs, and the black boxes denote the CDS. The vertical black lines indicate variations between prickly parent ‘PI 381159’ and prickleless parent ‘XQ23’. The red line indicates the transcription start site (TSS), while the green variant highlights the SNP at −735 bp (Chr6: 86555115). **b** Genotyping the SNP at −735 bp using the CAPS marker. A specific pair of primers was designed for this SNP to amplify the genomic DNA, which was then digested with the *SspI* restriction enzyme. PCR products of 152 bp and 239 bp represent homozygous genotypes identical to the prickly parent line ‘PI 381159’, whereas a 391 bp product indicates a homozygous genotype identical to the prickleless line ‘XQ23’. A mixture of 152 bp, 239 bp, and 391 bp products means heterozygous genotypes. M: marker DL2000. **c** Genotypic frequency of the −735 bp SNP within the eggplant germplasm population. ‘D’ denotes homozygous genotypes identical to the prickly parent line ‘PI 381159’; ‘R’ represents homozygous genotypes identical to the prickleless line ‘XQ23’; ‘H’ stands for heterozygous genotypes.

To explore genetic variations within the *SmLOG1* gene, specific primers (Table S7, see online supplementary material) were utilized to amplify the genomic sequences of *SmLOG1* from the parental lines ‘XQ23’ and ‘PI 381159’. A total of 7507 bp and 7503 bp were obtained, covering the promoter, gene and downstream regulatory region for ‘XQ23’ and ‘PI 381159’, respectively. Sequence analysis showed that variations between these two parents were confined to SNPs and InDels; notably, no variations were observed in the coding regions. SNPs were identified in the 5′-UTR region, while both SNPs and InDels were detected in the promoter and intron regions (Fig. 5a; Fig. S6, see online supplementary material). Specifically, two SNPs located at −574 bp and −735 bp, along with an 8 bp InDel spanning from −92 bp to −99 bp, were identified in the promoter region. In the 5′-UTR region, one SNP was found at +21 bp. In the intron region, four SNPs at +842 bp, +1192 bp, +2227 bp, and +2995 bp, as well as four 1 bp InDels at +275 bp, +3182 bp, +4364 bp, and +4951 bp, were detected. Given the *SmLOG1* gene’s tissue-specific expression predominantly in immature prickles, it is hypothesized that the SNPs or InDel within the promoter region could be causal variants, contributing to the differential expression patterns observed between the two parental lines.

To verify this hypothesis, we designed primers targeting the two SNPs and one InDel located in the promoter region (Table S7, see online supplementary material). Sanger sequencing was then performed to genotype these variants in 59 eggplant germplasms collected in our lab, which included 14 prickly and 45 prickleless eggplant accessions (Figs S7 and S8, see online supplementary material). The genotype–phenotype association analysis revealed that the SNP at −735 bp (G to A, located at base site 86 555 115 on chromosome 6) in the *SmLOG1* promoter exhibited a strong correlation with prickle trait variations. Specifically, each of the 14 prickly eggplant accessions possessed the ‘A’ allele, which matches the prickly parent line ‘PI 381159’, while the 45 prickleless accessions consistently exhibited the ‘G’ allele, aligning with the prickleless line ‘XQ23’ (Table S8 and Fig. S7, see online supplementary material). Following this discovery, we developed a CAPS marker based on this specific SNP (Fig. 5b; Table S7, see online supplementary material), then expanded our analysis to a broader germplasm collection comprising 190 eggplant varieties (Fig. 5c). The analysis showed that all 18 prickly eggplant accessions presented either the D (homozygous genotypes matching the prickly parent line ‘PI 381159’) or H (heterozygous) genotype. Conversely, of the 172 prickleless eggplant accessions, 166 (96.51%) possessed homozygous genotypes corresponding to the prickleless line ‘XQ23’, with only six accessions exhibiting the D genotype. Further examination reaffirmed the significant association of this SNP with prickle trait variations, as evidenced by a *P*-value of 1.90 × 10^−20^ from Fisher’s exact test. To understand the molecular mechanisms potentially influenced by this SNP, we analysed a 31 bp sequence surrounding it (15 bp upstream and 15 bp downstream), yielding sequences for prickly eggplant (GAGTTTTTTTTCAAT**A**TTTAAAATAGTTGAA) and prickleless eggplant (GAGTTTTTTTTCAAT**G**TTTAAAATAGTTGAA). Using JASPAR software [34], we predicted specific binding of the CPP (cysteine-rich polycomb-like protein) transcription factor family to the motif sequence (TTTTCAAT**A**TTTAAA or TTTCAAT**A**TTTAAAA) from prickly eggplant, while no transcription factors were predicted to bind to the sequence from prickleless eggplant (Table S9, see online supplementary material). It is well-documented that CPP proteins play a critical role in organ development and the control of cell division in plants [35]. For instance, *TSO1* modulates cell division activity, maintains a proper stem cell pool size, and balances cell proliferation with differentiation by negatively regulating *MYB3R1* in shoots and roots [36–38]. In summary, we conclude that the SNP located at −735 bp in the promoter region of *SmLOG1* is a highly likely causal variant affecting the gene’s expression levels and, consequently, prickle development.

## Discussion

### Distinct mechanisms underlie the development of prickles and trichomes

Prickles and trichomes, both being epidermal outgrowths, have led to speculation of a possible evolutionary link between them. The mainstream hypotheses suggest that prickles may have developed from glandular trichomes (GTs), with subsequent lignification transforming these soft structures into hard and sharp appendages [5], which is supported by morphological studies across various species, including grape [8], roses [9], and *Rubus* [10, 11]. Moreover, the upregulation of trichome-associated transcription factors, notably the MYB-bHLH-WD (MBW) complex and TTG2, in prickled roses compared to prickleless ones, suggests a shared genetic pathway, providing molecular evidence supporting the theory that prickles may evolve from trichomes [9, 39]. However, the origin of prickles challenges this theory; while trichomes arise from epidermal cells, prickles develop from multiple cell layers beneath the epidermis [12]. This fundamental difference is supported by observations that trichomes are still present in prickleless mutants in *Rubus* [10], rosa [14], and *Solanum viarum* [40]. Furthermore, transcription factors associated with trichome development show weak contribution to prickle formation in rose [13], indicating a possible divergence in their regulatory pathways.

In our previous study, we observed that both prickly and prickleless eggplant varieties exhibited a dense layer of trichomes, but without the presence of GTs on their surface, leading to the hypothesis that prickles might originate independently from GTs [15]. In this study, we genetically cloned the *SmLOG1* gene responsible for the formation of prickles in eggplant; subsequent gene knockout experiments demonstrated that *Smlog1* mutants lacked prickles without impacting trichome formation, thus suggesting a distinctive regulatory pathway. Considering the above evidence, it appears that while prickles and trichomes share common features as epidermal structures, their development is governed by markedly different gene regulatory networks. This, therefore, raises the question: Is there an intersection between the genetic pathways dictating prickle and trichome formation? Such inquiry warrants further exploration.

### Important role of *SmLOG1* and cytokinin in prickle development

Cytokinins are crucial in regulating meristematic tissue size and activity, thereby significantly affecting organ initiation and development [41, 42]. In plants, cytokinins primarily exist as isopentenyladenine (iP), trans-zeatin (tZ), and their nucleoside and nucleotide forms [43]. The family of *LOG* genes encode the cytokinin-activating enzyme that facilitate the final synthesis stage, converting inactive cytokinins into active forms [26]. This process involves the enzyme-mediated removal of ribose phosphate from isopentenyladenosine monophosphate (iPRMP) and trans-zeatin riboside monophosphate (tZRMP), yielding active cytokinins, iP and tZ [31]. Distinct *LOG* family members are known to regulate different plant tissues and organ developments. For instance, in rice, *OsLOG* is involved in the development of shoot apical meristems [31], while *LABA1*/*An-2* regulates the length of awns and yield [29, 30]; in Arabidopsis, *AtLOG3*, *AtLOG4*, and *AtLOG7* play roles in lateral root development [27]; in alfalfa, *MtLOG1* and *MtLOG2* are involved in the formation of root nodules [44].

In our study, the distinct expression patterns of *SmLOG1* observed in immature prickles indicates its crucial role in prickle morphogenesis. By enhancing cytokinin levels, the upregulated expression of cytokinin signaling-related genes such as *HK4* and *HP1* contributes to the formation and maintenance of meristematic regions essential for prickle development. The unique function attributed to *SmLOG1* in eggplant prickle morphogenesis distinguishes it from *LOG* gene counterparts in other plant species and organs, highlighting its unique involvement in prickle development. Further exploration of the specific pathways through which *SmLOG1* acts could reveal more about the relationship between cytokinin signaling and prickle formation, thereby expanding our understanding of plant developmental biology.

### The SNP in the promoter of *SmLOG1* as a crucial molecular marker in breeding

Promoter regions are fundamental to gene expression control, acting as crucial regulatory sequences that dictate the spatial, temporal, and quantitative aspects of a gene’s expression [45]. Variations such as SNPs and InDels within these regions can profoundly influence gene functionality by modulating expression levels or altering expression patterns, thereby affecting plant phenotypes significantly. For example, a specific SNP in the promoter of the *TGW2* gene in rice alters its expression, thereby impacting grain size and weight due to modified cell expansion in the glumes [46]. Similarly, two SNPs in the *MdMYB44* promoter influence apple fruit malate content by affecting gene expression and interaction with other proteins [47]. These instances underscore the strong relationship between promoter regions and gene function, highlighting their importance in both foundational and applied plant research.

In our study, we identified a SNP located at −735 bp within the promoter of the *SmLOG1* gene in eggplant, which is significantly associated with prickle phenotypes in 190 eggplant accessions. However, it is crucial to consider the genetic background of these accessions, as population structure can influence association results. Future studies should incorporate principal component analysis (PCA) or kinship analysis into the association analysis to better account for these effects. Additionally, we observed that six plants with the dominant alleles (D genotype) exhibited a prickleless phenotype, which suggests potential mutations in other loci of the *SmLOG1* gene or in different genes affecting prickle development. This anomaly underscores the complexity of genetic regulation in prickle formation and warrants further molecular investigation. We have also predicted specific binding of CPP transcription factors to the motif sequence containing this SNP in prickly eggplant, implying a regulatory role for these factors in the expression of *SmLOG1*. Further research is needed to confirm this interaction and to understand its implications for prickle morphogenesis. The identification of this SNP within the *SmLOG1* promoter highlights its value as a molecular marker for breeding applications. We have developed a CAPS marker based on this SNP. This marker could enable the development of prickleless eggplant varieties through marker-assisted selection, greatly simplifying the breeding process. By selecting for the prickleless-associated SNP variant early in the breeding cycle, breeders can more effectively propagate desirable traits, avoiding the slower and less accurate traditional phenotypic selection methods.

## Materials and methods

### Plant materials and phenotype measurement

The prickleless inbred line ‘XQ23’ (P_1_) was sourced from the Chinese seed market, while the prickly inbred line ‘PI 381159’ (P_2_) was ordered from the USDA Germplasm Resources Information Network. These lines, ‘XQ23’ (P_1_) and ‘PI 381159’ (P_2_), were selected as parental lines to generate F_1_ and F_2_ generations. Additionally, backcross populations were created by crossing the F_1_ generation with each parental line, producing BC_1_P_1_ (F_1_ × P_1_) and BC_1_P_2_ (F_1_ × P_2_), respectively. For phenotype analysis, the prickle phenotype of the parental, F_1_, F_2_, BC_1_P_1_, and BC_1_P_2_ populations were assessed at the flowering stage. The segregation ratio of the *PE* locus was tested by chi-squared analysis using the F_2_, BC_1_P_1_, and BC_1_P_2_ populations. Furthermore, this study included 190 eggplant accessions from a global collection for association analysis. Of these, 115 (60.53%) were acquired from the USDA-ARS Germplasm Resources Information Network (GRIN) with identifiers prefixed by ‘PI’ or ‘Grif’. The remaining 75 accessions were sourced from various commercial websites or the Chinese seed market. All plants were cultivated in the experimental field at Jiangsu Normal University, Xuzhou, China.

To investigate the structural features of prickle cells, we employed scanning electron microscopy (SEM) techniques. Initially, the prickles were stabilized in a fixative solution for two hours, then washed thrice with PBS. Afterward, the samples were further fixed in a 1% OsO4 solution for two additional hours, followed by another series of rinses in PBS. Subsequently, dehydration was performed using ethanol and isoamyl acetate for 15 minutes. Once dehydrated, the samples were dried using a Critical Point Dryer (Quorum Emitech, Kent, UK). They were then mounted on aluminum stubs and coated with gold palladium to ready them for imaging. The microscopic analysis was conducted using an SEM (Hitachi, Tokyo, Japan).

### Bulked segregant RNA-seq (BSR-seq)

BSR-seq analysis was conducted as previously described [48]. Within the F_2_ population, 30 individuals exhibiting the prickle phenotype and 30 individuals exhibiting the prickleless phenotype were randomly selected. RNA was extracted from each individual, then pooled equally to create prickly and prickleless pools. RNA sequencing was carried out on the Illumina NovaSeq6000 platform for both pools. Clean reads for each pool were generated using Trimmomatic software [49] and aligned to the eggplant reference genome HQ-1315 [50] using the STAR software [51], respectively. SNPs between the two pools were identified using Samtools software [52], with SNPs retained for further analysis if they met the criteria of a sequencing depth of ≥20, a mapping quality of ≥20, and a base quality of ≥20, utilizing an in-house Perl script. The SNP-index for each locus and the *△*(SNP-Index) between the pools were calculated. These *△*(SNP-Index) values and their 95% and 99% confidence intervals for each SNP locus were computed. The analysis employed a sliding window approach with a 400 Kb window and a 200 Kb step size to determine average *△*(SNP-Index) values and confidence intervals. Finally, *△*(SNP-Index) and its 95% and 99% statistical confidence intervals were graphically represented across the 12 chromosomes of the eggplant.

### Development of polymorphic markers and linkage mapping

The SNPs derived from BSR-seq were utilized to develop molecular markers, such as SNP (direct sequencing) and CAPS, aimed at narrowing the genetic interval for linkage mapping (Table S3, see online supplementary material). Sequence-specific primers for these markers were designed using Primer 3 software [53]. For the linkage mapping, a total of 145 individuals from the F_2_ segregating population were employed for preliminary mapping, while 1109 individuals were used for fine mapping.

### Phylogenetic analysis

The LOG proteins from *Arabidopsis*, eggplant, and rice (Table S4, see online supplementary material) were used to conduct phylogenetic analysis, following these steps. Initially, multiple sequence alignments of all proteins were performed utilizing ClustalW software [54]. Following this, a neighbor-joining phylogenetic tree with 1000 bootstrap replications was constructed using the MEGA software [55].

### Analysis of gene expression patterns

The transcriptome dataset from our previous study [15] was utilized to examine gene expression patterns across different tissue types. This dataset comprised three distinct tissues: immature prickles from the flower calyx of the prickly parent ‘PI 381159’ (immature prickles), matured prickles from the fruit calyx of the prickly parent ‘PI 381159’ (mature prickles), and the epidermis of the prickleless parent ‘XQ23’ (prickleless epidermis). Fragments per kilobase million (FPKM) values for *LOG* genes and cytokinin signaling-related genes were acquired for further analysis.

Various tissues (stem, leaf, root, flower, immature prickle, and mature prickle) from the parent lines ‘PI 381159’ or ‘XQ23’ were collected for qRT-PCR to explore the expression pattern of the *SmLOG1* gene. Total RNA was extracted using the OminiPlant RNA Kit (DNase I) (CWBiO, Beijing, China), followed by the synthesis of cDNA using the HiScript III All-in-one RT SuperMix Perfect for qPCR (Vazyme, Nanjing, China). The qRT-PCR experiments were conducted on the QuantStudioTM 6 Flex Real-Time PCR System (Thermo Fisher Scientific, Waltham, MA, USA) (Table S7, see online supplementary material). The normalization of expression levels was achieved by referencing the *SmAPRT* gene [56]. To evaluate the relative expression levels, the 2^-ΔΔCt^ method [57] was applied.

### Analysis of sequence polymorphism in the candidate gene

The candidate gene within the mapping interval was identified using the eggplant genome annotation file [50]. Gene-specific primers were designed based on the genomic sequence of the *SmLOG1* gene (Table S7, see online supplementary material). The genomic fragments of the *SmLOG1* gene from the two parental lines were obtained by overlapping PCR, conducted with PrimeSTAR GXL Premix (Takara, Japan). The resulting PCR products were sequenced and subsequently aligned with the reference genome to identify sequence variants.

### Vector construction and plant transformation

The CRISPR-Cas9 induced mutants were created as described previously [58]. Specifically, CRIPSR-Local [59] and CRISPRdirect [60] were employed for selecting target sites and predicting potential off-target sites. The selected two target sites each uniquely matched only once in both 20mer + PAM and 12mer + PAM searches, indicating high specificity. These target sites were incorporated into the forward and reverse PCR primers, respectively. The resulting PCR fragment amplified from pCBC-DT1T2_tomatoU6 was cloned into the pTX041 vector at the *BsaI* sites to create the final CRISPR-Cas9 vector (Table S7, see online supplementary material). This vector was then introduced into the prickly eggplant ‘PI 381159’ following previously described methods [61]. The mutants were genotyped using PCR amplification followed by Sanger sequencing.

### Accession numbers

The *SmLOG1* and *Smlog1* genomic sequences have been deposited in the National Center for Biotechnology Information (NCBI) with the accession numbers PP537947 and PP537948, respectively. The BSR-seq data have been deposited in the NCBI Sequence Read Archive (SRA) under the BioProject accession number PRJNA1091114. The RNA-seq data utilized in this research, which was published earlier, is available at the NCBI SRA database with the BioProject accession number PRJNA695792.

## Supplementary Material

Web_Material_uhae134

## Data Availability

All relevant data can be found within the article and its supporting materials.

## References

[1] Yang X, Cheng Y-F, Deng C. et al. Comparative transcriptome analysis of eggplant (*Solanum melongena* L.) and Turkey berry (*Solanum torvum* Sw.): phylogenomics and disease resistance analysis. BMC Genomics. 2014;15:412 24885385 10.1186/1471-2164-15-412 PMC4070557

[2] Page A, Gibson J, Meyer RS. et al. Eggplant domestication: pervasive gene flow, feralisation and transcriptomic divergence. Mol Biol Evol. 2019;36:1359–72 31039581 10.1093/molbev/msz062

[3] Miyatake K, Saito T, Nunome T. et al. Fine mapping of a major locus representing the lack of prickles in eggplant revealed the availability of a 0.5-kb insertion/deletion for marker-assisted selection. Breed Sci. 2020;70:438–48 32968346 10.1270/jsbbs.20004 PMC7495204

[4] Coverdale TC . Defence emergence during early ontogeny reveals important differences between spines, thorns and prickles. Ann Bot. 2019;124:iii–v 10.1093/aob/mcz189 PMC6943688 31904092

[5] Pei H, Wu Y, Wu W. et al. A review of the types, functions and regulatory mechanisms of plant spines. Plant Sci. 2024;341:112010 38309475 10.1016/j.plantsci.2024.112010

[6] Simpson MG . 9 - Plant Morphology. In: Simpson MG (ed.), Plant Systematics. 2nd edn. San Diego: Academic Press, 2010, 451–513

[7] Wang X, Shen C, Meng P. et al. Analysis and review of trichomes in plants. BMC Plant Biol. 2021;21:70 33526015 10.1186/s12870-021-02840-x PMC7852143

[8] Ma ZY, Wen J, Ickert-Bond SM. et al. Morphology, structure, and ontogeny of trichomes of the grape genus (Vitis, Vitaceae). Front Plant Sci. 2016;7:704 27252720 10.3389/fpls.2016.00704 PMC4879774

[9] Swarnkar MK, Kumar P, Dogra V. et al. Prickle morphogenesis in rose is coupled with secondary metabolite accumulation and governed by canonical MBW transcriptional complex. Plant Direct. 2021;5:e00325 34142001 10.1002/pld3.325 PMC8204143

[10] Khadgi A, Weber CA. Morphological characterization of prickled and prickle-free Rubus using scanning electron microscopy. HortScience. 2020;55:676–83

[11] Kellogg AA, Branaman TJ, Jones NM. et al. Morphological studies of developing Rubus prickles suggest that they are modified glandular trichomes. Botany. 2011;89:217–26

[12] Zhou N, Simonneau F, Thouroude T. et al. Morphological studies of rose prickles provide new insights. Hort Res. 2021;8:221 10.1038/s41438-021-00689-7 PMC8460668 34556626

[13] Zhou NN, Tang KX, Jeauffre J. et al. Genetic determinism of prickles in rose. TAG Theoretical Applied Genet. 2020;133:3017–35 10.1007/s00122-020-03652-7 32734323

[14] Zhang Y, Zuo M, Li R. et al. Morphology, structure and development of glandular prickles in the genus Rosa. Sci Hortic. 2024;326:112763

[15] Zhang L, Sun H, Xu T. et al. Comparative transcriptome analysis reveals key genes and pathways involved in prickle development in eggplant. Genes. 2021;12:341 33668977 10.3390/genes12030341 PMC7996550

[16] Huang X, Yi P, Liu Y. et al. RrTTG1 promotes fruit prickle development through an MBW complex in Rosa roxburghii. Front Plant Sci. 2022;13:939270 36105707 10.3389/fpls.2022.939270 PMC9465040

[17] Pattanaik S, Patra B, Singh SK. et al. An overview of the gene regulatory network controlling trichome development in the model plant, Arabidopsis. Front Plant Sci. 2014;5:00259 10.3389/fpls.2014.00259 PMC4071814 25018756

[18] Li S, He Y, Li D. et al. Fine mapping an AUXIN RESPONSE FACTOR, SmARF18, as a candidate gene of the PRICKLE LOCUS that controls prickle absence/presence on various organs in eggplant (*Solanum melongena* L.). Sci Hortic. 2024;327:112874

[19] Doganlar S, Frary A, Daunay MC. et al. Conservation of gene function in the solanaceae as revealed by comparative mapping of domestication traits in eggplant. Genetics. 2002;161:1713–26 12196413 10.1093/genetics/161.4.1713 PMC1462228

[20] Frary A, Frary A, Daunay M-C. et al. QTL hotspots in eggplant (*Solanum melongena*) detected with a high resolution map and CIM analysis. Euphytica. 2014;197:211–28

[21] Portis E, Barchi L, Toppino L. et al. QTL mapping in eggplant reveals clusters of yield-related loci and orthology with the tomato genome. PLoS One. 2014;9:e89499 24586828 10.1371/journal.pone.0089499 PMC3931786

[22] Portis E, Cericola F, Barchi L. et al. Association mapping for fruit, plant and leaf morphology traits in eggplant. PLoS One. 2015;10:e0135200 26284782 10.1371/journal.pone.0135200 PMC4540451

[23] Mangino G, Plazas M, Vilanova S. et al. Performance of a set of eggplant (*Solanum melongena*) lines with introgressions from its wild relative *S. incanum* under open field and Screenhouse conditions and detection of QTLs. Agronomy. 2020;10:10040467

[24] Wei Q, Wang W, Hu T. et al. Construction of a SNP-based genetic map using SLAF-Seq and QTL analysis of morphological traits in eggplant. Front Genet. 2020;11:178 32218801 10.3389/fgene.2020.00178 PMC7078336

[25] Qian Z, Zhang B, Chen H. et al. Identification of quantitative trait loci controlling the development of prickles in eggplant by genome re-sequencing analysis. Front Plant Sci. 2021;12:731079 10.3389/fpls.2021.731079 PMC8457335 34567042

[26] Chen L, Jameson GB, Guo Y. et al. The LONELY GUY gene family: from mosses to wheat, the key to the formation of active cytokinins in plants. Plant Biotechnol J. 2022;20:625–45 35108444 10.1111/pbi.13783 PMC8989509

[27] Kuroha T, Tokunaga H, Kojima M. et al. Functional analyses of LONELY GUY cytokinin-activating enzymes reveal the importance of the direct activation pathway in Arabidopsis. Plant Cell. 2009;21:3152–69 19837870 10.1105/tpc.109.068676 PMC2782294

[28] Tokunaga H, Kojima M, Kuroha T. et al. Arabidopsis lonely guy (LOG) multiple mutants reveal a central role of the LOG-dependent pathway in cytokinin activation. Plant J. 2012;69:355–65 22059596 10.1111/j.1365-313X.2011.04795.x

[29] Hua L, Wang DR, Tan L. et al. LABA1, a domestication gene associated with long, barbed awns in wild rice. Plant Cell. 2015;27:1875–88 26082172 10.1105/tpc.15.00260 PMC4531357

[30] Gu B, Zhou T, Luo J. et al. An-2 encodes a cytokinin synthesis enzyme that regulates awn length and grain production in rice. Mol Plant. 2015;8:1635–50 26283047 10.1016/j.molp.2015.08.001

[31] Kurakawa T, Ueda N, Maekawa M. et al. Direct control of shoot meristem activity by a cytokinin-activating enzyme. Nature. 2007;445:652–5 17287810 10.1038/nature05504

[32] Kieber JJ, Schaller GE. Cytokinins. Arabidopsis Book. 2014;12:e0168 24465173 10.1199/tab.0168 PMC3894907

[33] Pils B, Heyl A. Unraveling the evolution of cytokinin signaling. Plant Physiol. 2009;151:782–91 19675156 10.1104/pp.109.139188 PMC2754637

[34] Castro-Mondragon JA, Riudavets-Puig R, Rauluseviciute I. et al. JASPAR 2022: the 9th release of the open-access database of transcription factor binding profiles. Nucleic Acids Res. 2022;50:D165–73 34850907 10.1093/nar/gkab1113 PMC8728201

[35] Yang Z, Gu S, Wang X. et al. Molecular evolution of the CPP-like gene family in plants: insights from comparative genomics of Arabidopsis and Rice. J Mol Evol. 2008;67:266–77 18696028 10.1007/s00239-008-9143-z

[36] Liu Z, Running MP, Meyerowitz EM. TSO1 functions in cell division during Arabidopsis flower development. Development. 1997;124:665–72 9043081 10.1242/dev.124.3.665

[37] Hauser BA, He JQ, Park SO. et al. TSO1 is a novel protein that modulates cytokinesis and cell expansion in Arabidopsis. Development. 2000;127:2219–26 10769245 10.1242/dev.127.10.2219

[38] Wang W, Sijacic P, Xu P. et al. Arabidopsis TSO1 and MYB3R1 form a regulatory module to coordinate cell proliferation with differentiation in shoot and root. Proc Natl Acad Sci USA. 2018;115:E3045–54 29535223 10.1073/pnas.1715903115 PMC5879663

[39] Hibrand Saint-Oyant L, Ruttink T, Hamama L. et al. A high-quality genome sequence of *Rosa chinensis* to elucidate ornamental traits. Nature Plants. 2018;4:473–84 29892093 10.1038/s41477-018-0166-1 PMC6786968

[40] Pandey S, Goel R, Bhardwaj A. et al. Transcriptome analysis provides insight into prickle development and its link to defense and secondary metabolism in *Solanum viarum* Dunal. Sci Rep. 2018;8:17092 30459319 10.1038/s41598-018-35304-8 PMC6244164

[41] Osugi A, Sakakibara H. Q&A: how do plants respond to cytokinins and what is their importance? BMC Biol. 2015;13:102 26614311 10.1186/s12915-015-0214-5 PMC4662812

[42] Hnatuszko-Konka K, Gerszberg A, Weremczuk-Jeżyna I. et al. Cytokinin signaling and de novo shoot organogenesis. Genes. 2021;12:265 33673064 10.3390/genes12020265 PMC7917986

[43] Hai NN, Chuong NN, Tu NHC. et al. Role and regulation of cytokinins in plant response to drought stress. Plan Theory. 2020;9:422 10.3390/plants9040422 PMC7238249 32244272

[44] Mortier V, Wasson A, Jaworek P. et al. Role of LONELY GUY genes in indeterminate nodulation on *Medicago truncatula*. New Phytol. 2014;202:582–93 24443934 10.1111/nph.12681

[45] Villao-Uzho L, Chávez-Navarrete T, Pacheco-Coello R. et al. Plant promoters: their identification, characterization, and role in gene regulation. Genes. 2023;14:1226 37372407 10.3390/genes14061226 PMC10298551

[46] Ruan B, Shang L, Zhang B. et al. Natural variation in the promoter of TGW2 determines grain width and weight in rice. New Phytol. 2020;227:629–40 32167575 10.1111/nph.16540

[47] Jia D, Wu P, Shen F. et al. Genetic variation in the promoter of an R2R3−MYB transcription factor determines fruit malate content in apple (*Malus domestica* Borkh.). Plant Physiol. 2021;186:549–68 33624810 10.1093/plphys/kiab098 PMC8154052

[48] Zhang L, Qian J, Han Y. et al. Alternative splicing triggered by the insertion of a CACTA transposon attenuates LsGLK and leads to the development of pale-green leaves in lettuce. Plant J. 2022;109:182–95 34724596 10.1111/tpj.15563

[49] Bolger AM, Lohse M, Usadel B. Trimmomatic: a flexible trimmer for Illumina sequence data. Bioinformatics. 2014;30:2114–20 24695404 10.1093/bioinformatics/btu170 PMC4103590

[50] Wei Q, Wang J, Wang W. et al. A high-quality chromosome-level genome assembly reveals genetics for important traits in eggplant. Hortic Res. 2020;7:153 33024567 10.1038/s41438-020-00391-0 PMC7506008

[51] Dobin A, Davis CA, Schlesinger F. et al. STAR: ultrafast universal RNA-seq aligner. Bioinformatics. 2013;29:15–21 23104886 10.1093/bioinformatics/bts635 PMC3530905

[52] Li H, Handsaker B, Wysoker A. et al. The sequence alignment/map format and SAMtools. Bioinformatics. 2009;25:2078–9 19505943 10.1093/bioinformatics/btp352 PMC2723002

[53] Untergasser A, Nijveen H, Rao X. et al. Primer3Plus, an enhanced web interface to Primer3. Nucleic Acids Res. 2007;35:W71–4 17485472 10.1093/nar/gkm306 PMC1933133

[54] Thompson JD, Higgns DG, Gibson Toby J. CLUSTAL W: improving the sensitivity of progressive multiple sequence alignment through sequence weighting, position-specific gap penalties and weight matrix choice. Nucleic Acids Res. 1994;22:4673–80 7984417 10.1093/nar/22.22.4673 PMC308517

[55] Kumar S, Stecher G, Tamura K. MEGA7: molecular evolutionary genetics analysis version 7.0 for bigger datasets. Mol Biol Evol. 2016;33:1870–4 27004904 10.1093/molbev/msw054 PMC8210823

[56] Gantasala NP, Papolu PK, Thakur PK. et al. Selection and validation of reference genes for quantitative gene expression studies by real-time PCR in eggplant (*Solanum melongena* L). BMC Res Notes. 2013;6:312 23919495 10.1186/1756-0500-6-312 PMC3750715

[57] Livak KJ, Schmittgen TD. Analysis of relative gene expression data using real-time quantitative PCR and the 2(-Delta Delta C(T)) method. Methods. 2001;25:402–8 11846609 10.1006/meth.2001.1262

[58] Deng L, Wang H, Sun C. et al. Efficient generation of pink-fruited tomatoes using CRISPR/Cas9 system. J Genet Genomics. 2018;45:51–4 29157799 10.1016/j.jgg.2017.10.002

[59] Liu H, Ding Y, Zhou Y. et al. CRISPR-P 2.0: an improved CRISPR-Cas9 tool for genome editing in plants. Mol Plant. 2017;10:530–2 28089950 10.1016/j.molp.2017.01.003

[60] Naito Y, Hino K, Bono H. et al. CRISPRdirect: software for designing CRISPR/Cas guide RNA with reduced off-target sites. Bioinformatics. 2015;31:1120–3 25414360 10.1093/bioinformatics/btu743 PMC4382898

[61] Khatun M, Borphukan B, Alam I. et al. An improved Agrobacterium mediated transformation and regeneration protocol for successful genetic engineering and genome editing in eggplant. Sci Hortic. 2021;293:110716

