# Supplementary material for: PE (Prickly Eggplant) encoding a cytokinin-activating enzyme responsible for the formation of prickles in eggplant
Source: Hortic Res. 2024 May 10;11(7):uhae134. doi: 10.1093/hr/uhae134 (PMC11226868; doi:10.1093/hr/uhae134)
Supplement: Web_Material_uhae134 [file web_material_uhae134.zip › Supplementary_figures - small.docx]

###### Supplementary Figures:


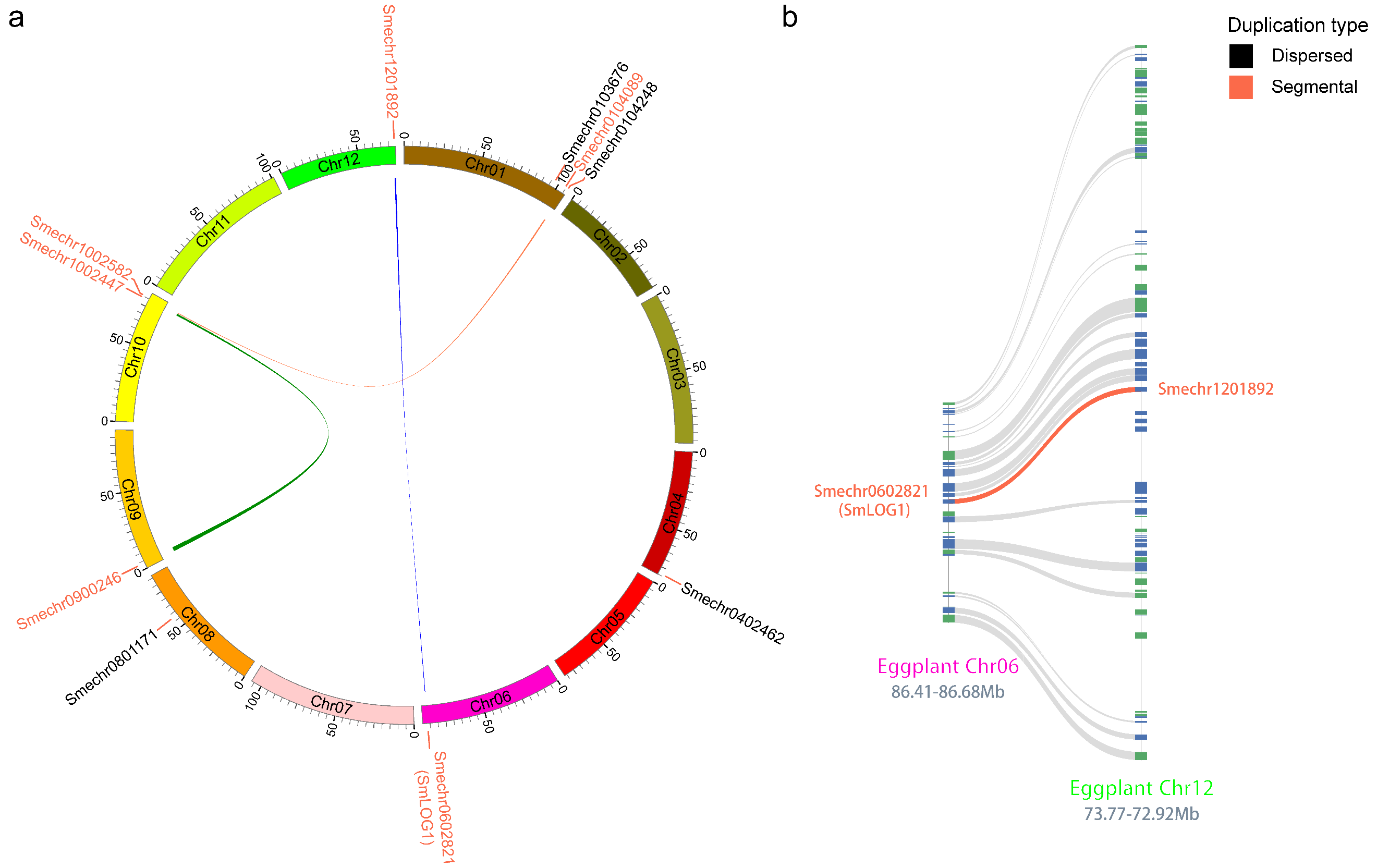


**Figure S1. Syntenic relationships of** ***SmLOG* genes**

(a) Chromosomal distribution and synteny of *SmLOG* genes. The synteny analysis identified three pairwise syntenic blocks containing six *SmLOG* genes, including *SmLOG1* and *Smechr1201892*. Each eggplant chromosome is represented in a distinct color. Colored lines indicate syntenic blocks. Genes within syntenic blocks are marked in red. (b) Detailed view of the microsynteny in blocks containing *SmLOG1* and *Smechr1201892* (specifically Chr06: 86.41-86.68 Mb for *SmLOG1*, and Chr12:72.92-73.77 Mb for *Smechr1201892*). Genes are color-coded blue and green to indicate forward and reverse orientations, respectively. The syntenic pair of *SmLOG1* and *Smechr1201892* is connected by a red line, while other syntenic pairs are connected by gray lines. MCscanX software was utilized to identify syntenic blocks within the eggplant genome. Circos software (v0.69) was employed to illustrate the segmental duplications of *SmLOG* genes. MCscan (Python version) was used to visualize microsynteny among blocks containing *SmLOG1* and *Smechr1201892*.


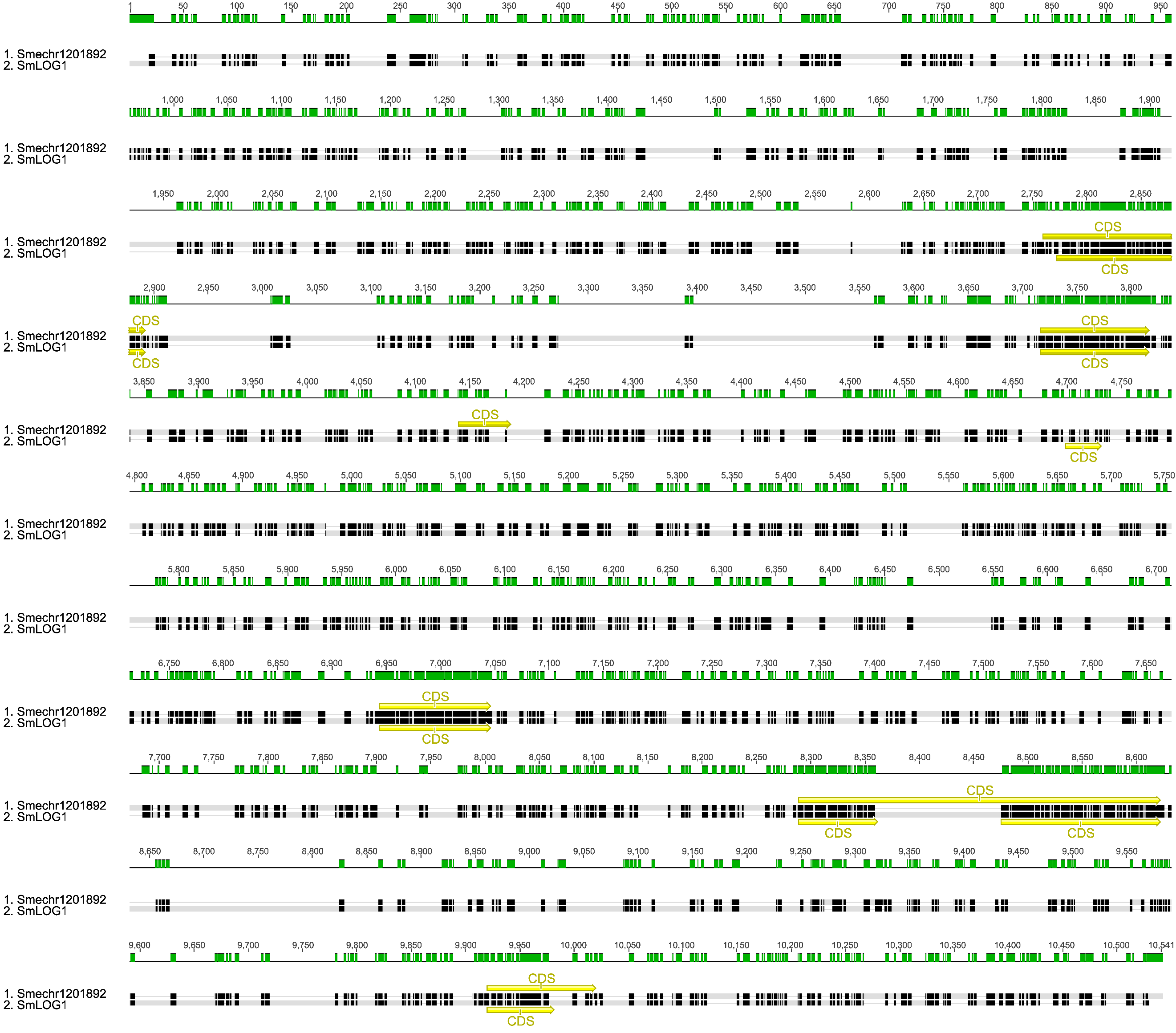


**Figure S2. Alignment of the genomic sequences of** ***SmLOG1* and** ***Smechr1201892***

The Geneious software was utilized for aligning the genomic sequences of *SmLOG1* and *Smechr1201892*, as well as for creating the schematic diagram of this alignment. The histogram at the top illustrates the sequence identity between the *SmLOG1* and *Smechr1201892* genomic sequences, with green denoting 100% identity. A solid black bar signifies sections where the *SmLOG1* and *Smechr1201892* genomic sequences are identical, while a solid grey bar indicates regions with gaps in the alignment of *SmLOG1* and *Smechr1201892*. Additionally, arrows positioned above *Smechr1201892* or below *SmLOG1* denote the coding sequences (CDS), with their orientation reflecting the respective directions.


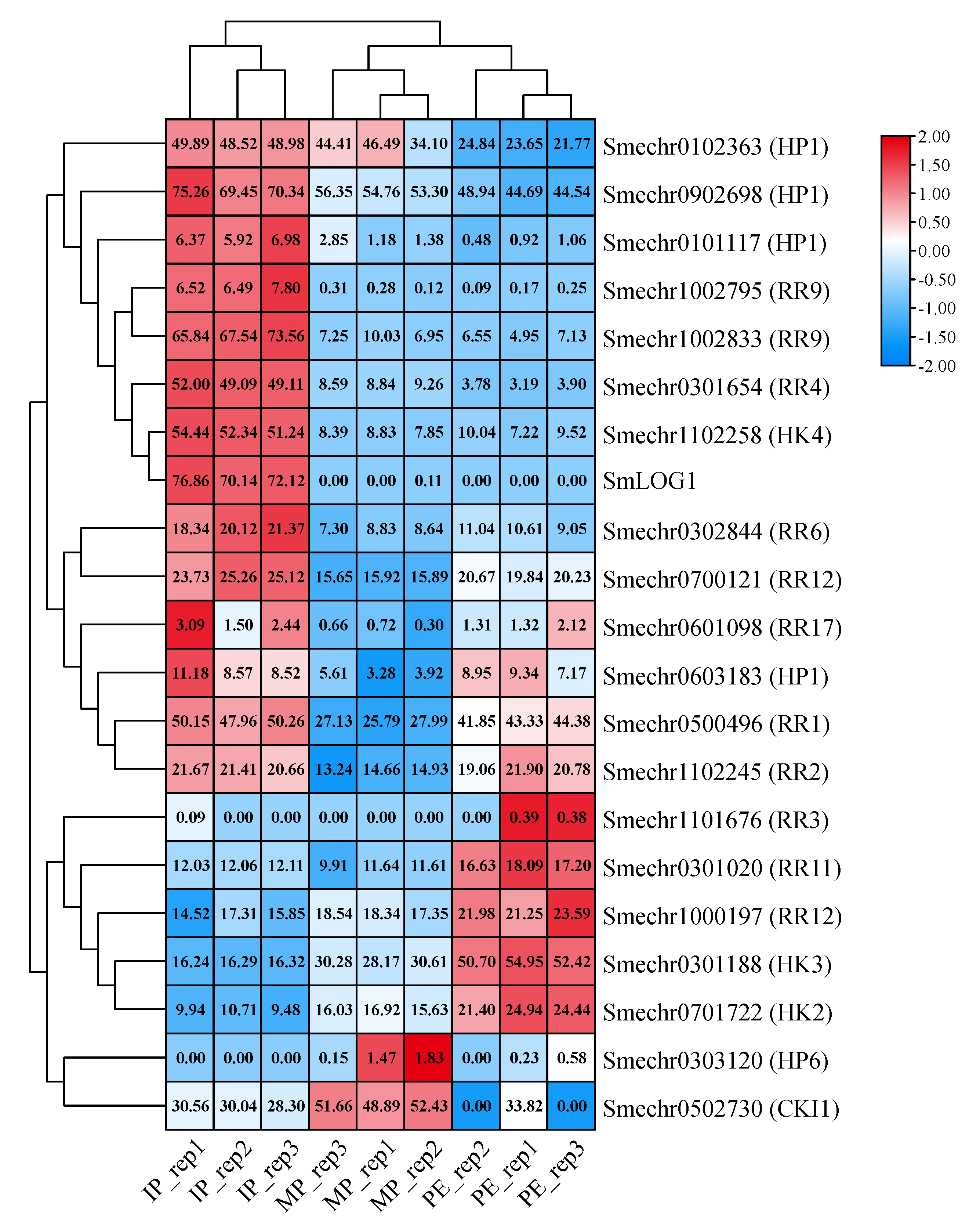


**Figure S3. Heatmap depicting the expression patterns of cytokinin signaling-related genes in immature prickles, mature prickles, and prickleless epidermis**

IP, MP, and PE correspond to immature prickles, mature prickles, and prickleless epidermis, respectively. Genes expressed in at least three samples (nonzero FPKM values) were selected for heatmap analysis. The heatmap displays normalized log_2_(FPKM+1) values. Each tissue type is represented by three biological replicates, and the corresponding FPKM values are indicated in each cell of the heatmap.


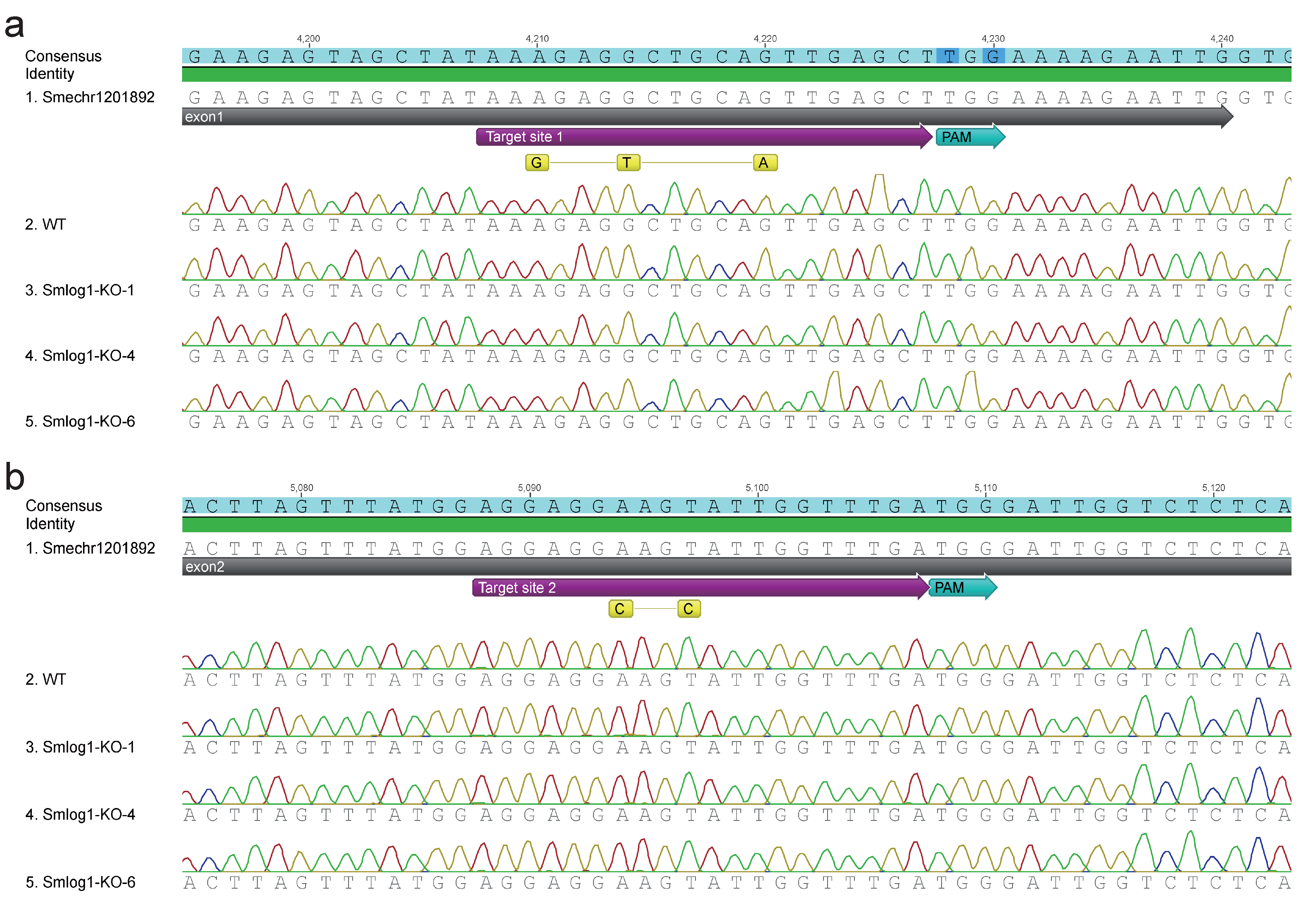


**Figure S4. Sanger sequencing at target sites** **corresponding to *Smechr1201892***

Direct sequencing of PCR product chromatograms from two target sites corresponding to *Smechr1201892* in the wild type (WT, prickly eggplant 'PI381159') and three representative homozygous T_1_ plants (*Smlog1-KO-1*, *Smlog1-KO-4*, and *Smlog1-KO-6*). Purple arrows indicate the two target sites matching the first and second exons of *Smechr1201892*. Yellow boxes highlight mismatches between the target sites and *Smechr1201892*. Cyan arrows represent the protospacer adjacent motifs (PAM).


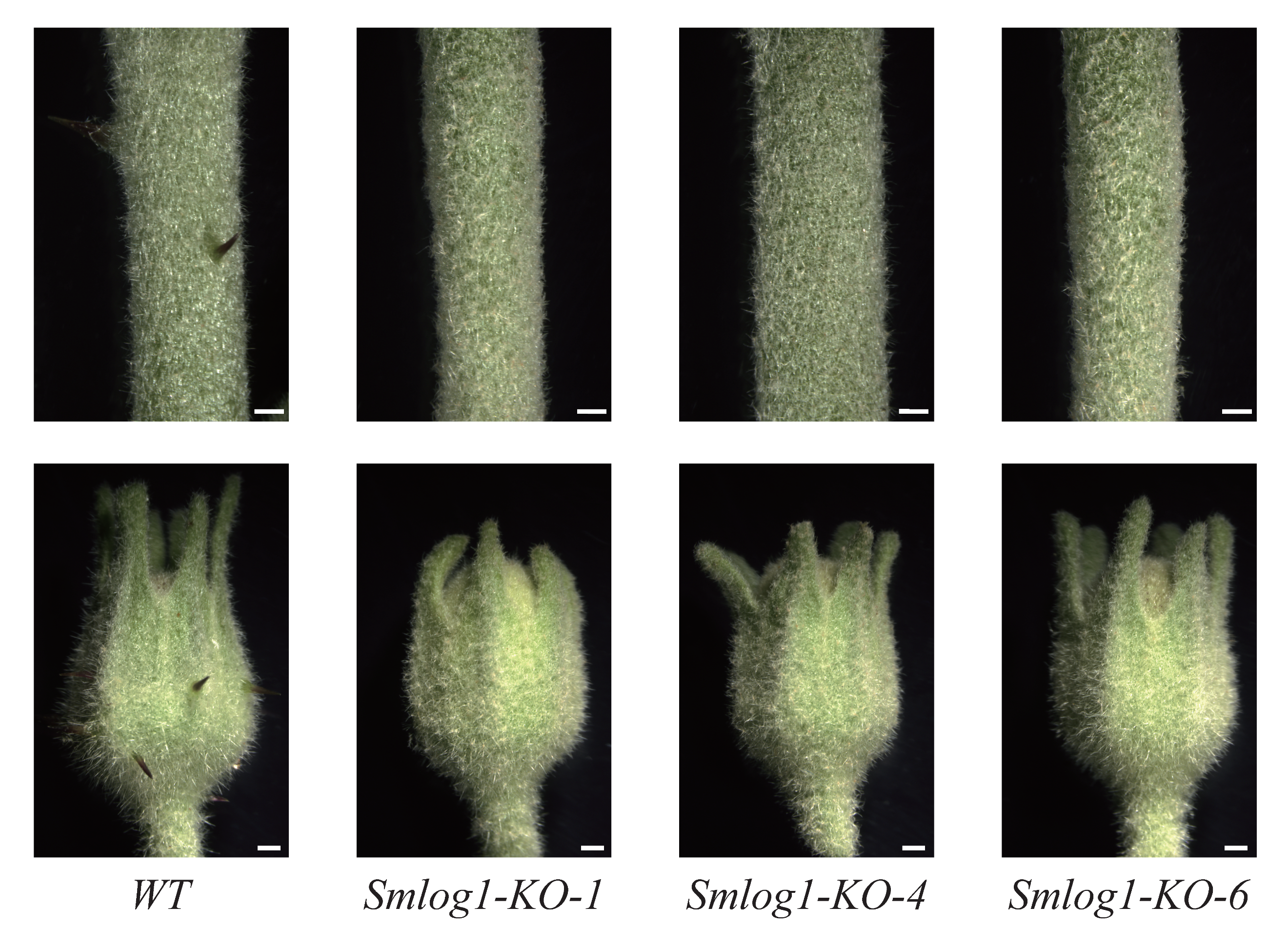


**Figure S5. Stereo microscope images of stems and buds from wild type and *SmLOG1* knockout mutants**

Images showcase tissues from the wild type (WT, prickly eggplant 'PI381159') and three homozygous T_1_ plants (*Smlog1-KO-1*, *Smlog1-KO-4*, and *Smlog1-KO-6*). Observations were made using a Leica S8 APO stereo microscope (Leica Microsystems, Germany), and photographs were captured with a Leica DMC4500 digital camera. Images were processed using Leica Application Suite V4.10 software (Leica Microsystems, Germany). Scale bar: 1 mm.


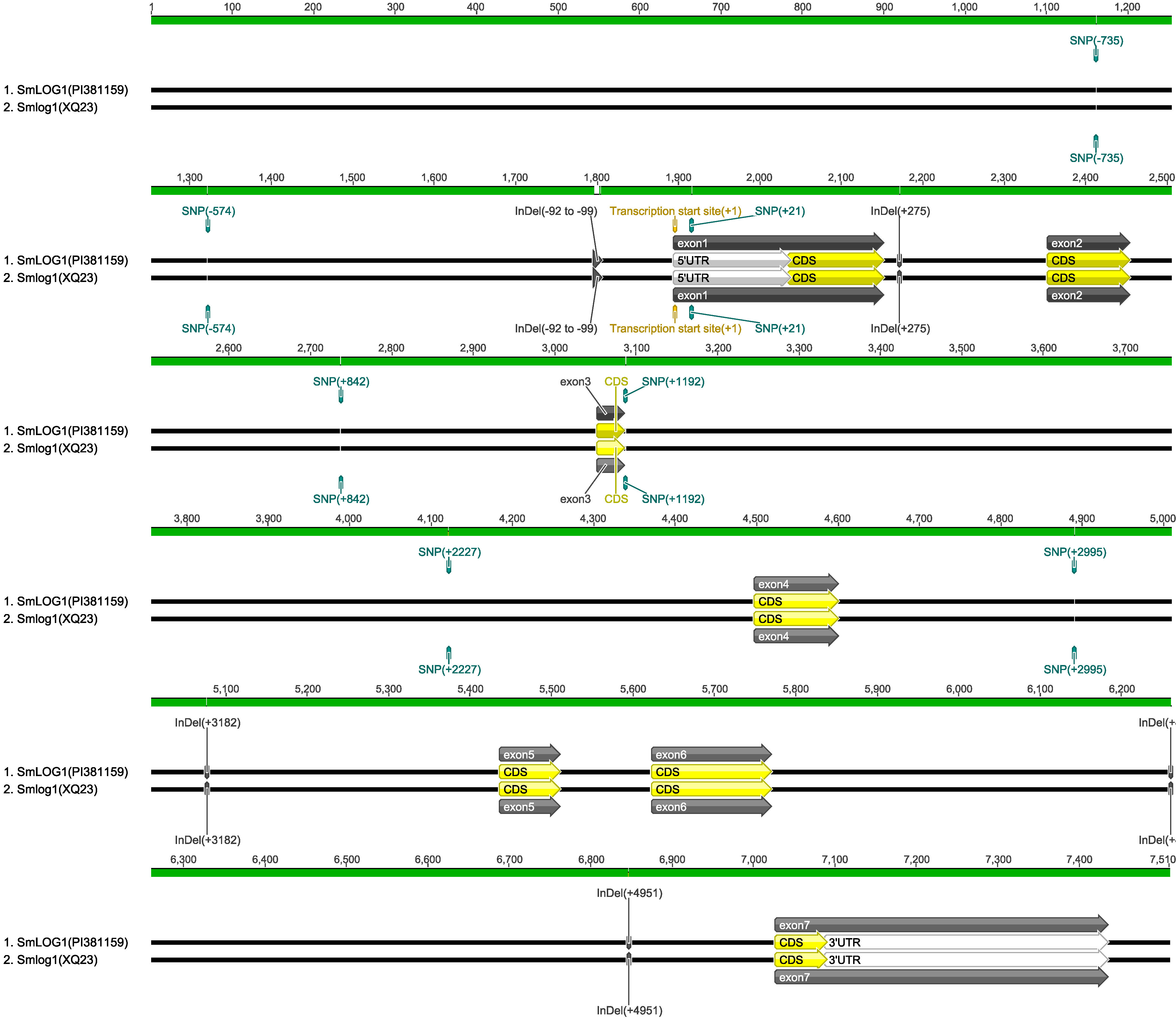


**Figure S6. Schematic representation of sequence variations of *SmLOG1* between prickly eggplant 'PI381159' and prickleless eggplant 'XQ23'**

The Geneious software was utilized for aligning the genomic sequences of *SmLOG1* and *Smlog1*, as well as for creating the schematic diagram of this alignment. The histogram at the top illustrates the sequence identity, with green denoting 100% identity. A solid black bar signifies identical sequences, while a solid grey bar indicates mismatched sequences.

**
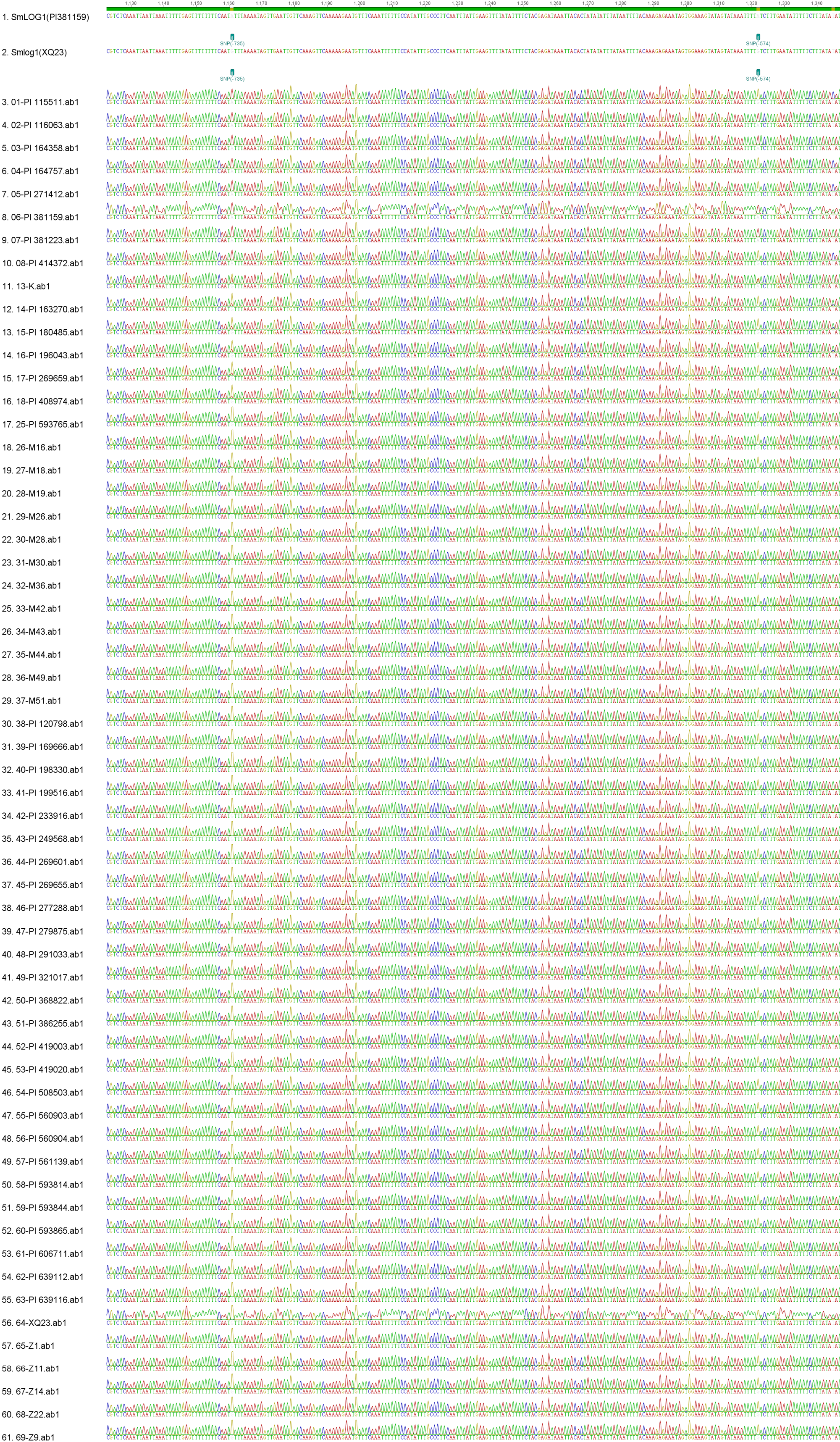
**

**Figure S7. Direct sequencing of PCR product chromatograms from the two SNPs (-574 and -735) in promoter region of the *SmLOG1* gene across 59 eggplant germplasms**

The Geneious software was employed for sequence alignment and for generating the corresponding schematic diagrams. The histogram displayed at the top highlights sequence similarity, with areas of 100% identity marked in green. For the SNP located at -735, each of the 14 prickly eggplant accessions possessed the 'A' allele, which matches the prickly parent line 'PI381159', while the 45 prickleless accessions consistently exhibited the 'G' allele, aligning with the prickleless line 'XQ23'.


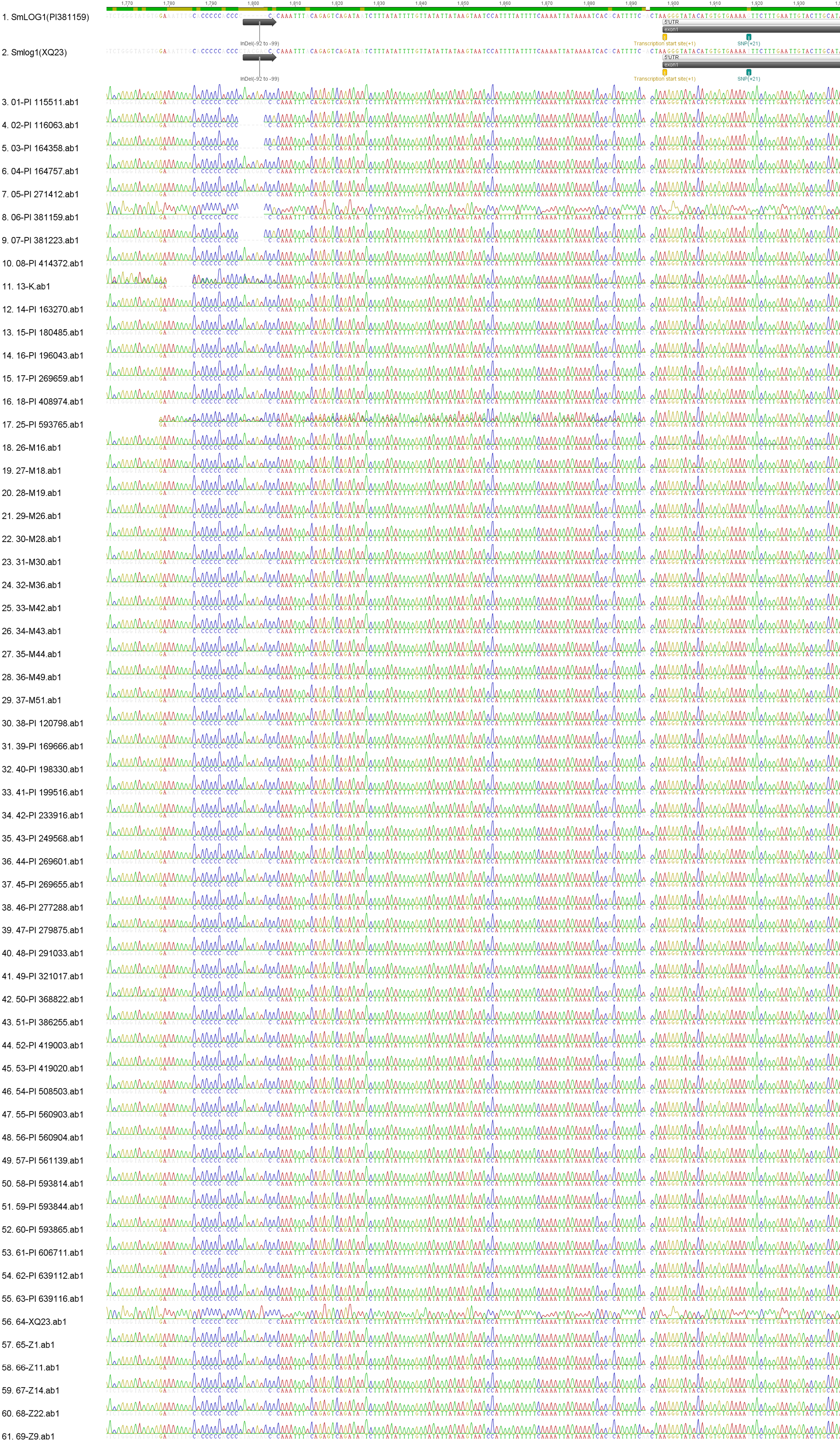
**Figure S8. Direct sequencing of PCR product chromatograms from the InDel (-92 bp to -99 bp) in the promoter and SNP (+21) in the 5’-UTR of the *SmLOG1* gene across 59 eggplant germplasms**

The Geneious software was employed for sequence alignment and for generating the corresponding schematic diagrams. The histogram displayed at the top highlights sequence similarity, with areas of 100% identity marked in green.
